# Prevalence of Intracranial Hemorrhage after Blunt Head Trauma in Patients on Pre-injury Dabigatran

**DOI:** 10.5811/westjem.2017.5.33092

**Published:** 2017-07-14

**Authors:** James A. Chenoweth, M. Austin Johnson, Laura Shook, Mark E. Sutter, Daniel K. Nishijima, James F. Holmes

**Affiliations:** * University of California Davis, School of Medicine, Department of Emergency Medicine, Davis, California; † Veterans Affairs Northern California, Mather Medical Center, Mather, California; ‡ University of California Davis, School of Medicine, Davis, California

## Abstract

**Introduction:**

Dabigatran etexilate was the first direct-acting oral anticoagulant approved in the United States. The prevalence of intracranial hemorrhage after blunt head trauma in patients on dabigatran is currently unknown, complicating adequate ability to accurately compare the risks and benefits of dabigatran to alternative anticoagulants. We aimed to determine the prevalence of intracranial hemorrhage for patients on dabigatran presenting to a Level I trauma center.

**Methods:**

This is a retrospective observational study of adult patients on dabigatran who presented to a Level I trauma center and received cranial computed tomography (CT) following blunt head trauma. Patients who met inclusion criteria underwent manual chart abstraction. Our primary outcome was intracranial hemorrhage on initial cranial CT.

**Results:**

We included a total of 33 eligible patient visits for analysis. Mean age was 74.8 years (SD 11.2, range 55–91). The most common cause of injury was ground-level fall (n = 22, 66.7%). One patient (3.0%, 95% confidence interval [CI] 0.[1–15.8%]) had intracranial hemorrhage on cranial CT. No patients (0%, 95% CI [0–8.7%]) required neurosurgical intervention. One in-hospital death occurred from infection.

**Conclusion:**

To our knowledge, this is the first study to evaluate the prevalence of intracranial hemorrhage after blunt head trauma for patients on dabigatran presenting to the emergency department, including those not admitted. The intracranial hemorrhage prevalence in our study is similar to previous reports for patients on warfarin. Further studies are needed to determine if the prevalence of intracranial hemorrhage seen in our patient population is true for a larger patient population in more diverse clinical settings.

## INTRODUCTION

Dabigatran etexilate is a direct thrombin inhibitor first approved in October 2010 for primary prevention of stroke in patients with atrial fibrillation.^1^ It is the first of several direct-acting oral anticoagulants (DOAC) to market. These agents have gained popularity due to the simplicity of dosing and the fact that they do not require routine laboratory testing.^2^ In fact, a recent cohort study of patients started on anticoagulation medications for atrial fibrillation indicated that up to 62% were prescribed a DOAC.^3^

Each year traumatic brain injury results in approximately 1.4 million emergency department (ED) visits at an annual cost of U.S. $60 billion.^4^ An increasing proportion of these patients are elderly and taking anticoagulant medications.^5^ Patients on pre-injury warfarin or clopidogrel have significant risk of traumatic intracranial hemorrhage, even in cases with low-impact mechanisms of injury.^6^

In addition to the increased risk of intracranial hemorrhage, pre-injury use of warfarin also significantly increases mortality in elderly patients after head trauma. Early data suggest that this may not be the case for patients on DOACs, although the data for dabigatran is mixed.^10^–^12^ Additionally, management of trauma patients on dabigatran is especially challenging due to questions regarding coagulation testing and reversal strategies.^13^

Despite Food and Drug Administration approval six years ago, the true prevalence of intracranial hemorrhage after head trauma for patients on dabigatran is still unknown. Furthermore, there is a paucity of evidence regarding monitoring of the level of anticoagulation in trauma victims on dabigatran. In this study we aimed to determine the prevalence of intracranial hemorrhage for patients on pre-injury dabigatran presenting to a Level I trauma center after blunt head trauma.

## METHODS

### Study Setting and Design

This is a retrospective observational study of all patients presenting to a university-based, urban Level 1 trauma center between November 1, 2010 and February 28, 2015. The study was approved by the site’s institutional review board.

### Selection of Participants

All patients who received cranial computed tomography (CT) as part of their ED evaluation and were reported to be on dabigatran during the study period were evaluated for study inclusion. Inclusion criteria were as follows: 1) patient reported head trauma or physical examination findings of head trauma were documented in the ED history and physical examination, and 2) patient was over the age of 18 years at the time of ED presentation. We excluded from the final analysis prisoners, patients who were transferred from an outside hospital, and pregnant patients.

### Data Collection and Processing

Manual chart abstraction from the electronic medical record was performed by a single abstractor for all patients who met inclusion criteria. Standard chart review methodology was followed for all data abstraction.^14^ Investigators agreed upon all inclusion/exclusion criteria and definitions prior to chart abstraction. All patient baseline data were abstracted prior to abstraction of CT results. A second abstractor reviewed approximately 20% of charts to measure interrater reliability for the presence of trauma (inclusion/exclusion criteria), intracranial hemorrhage on cranial CT, and the initial Glasgow Coma Scale (GCS) score.

Baseline factors including age, sex, and indication for anticoagulation (atrial fibrillation, deep venous thrombosis, pulmonary embolism, other, or unknown) were recorded. Data regarding the mechanism of injury, initial GCS score, international normalized ratio (INR), and activated thromboplastin time were abstracted from the electronic medical record. For all patients admitted to the hospital we calculated an abbreviated injury severity score (ISS).^15^

Population Health Research Capsule

What do we already know about this issue?

Clinical trial data suggests an improved bleeding profile for dabigatran when compared to warfarin. There are limited data regarding the intracranial hemorrhage risk after head trauma.

What was the research question?

What is the prevalence of intracranial hemorrhage after blunt head trauma for patients on dabigatran?

What was the major finding of the study?

Dabigatran appears to have a similar prevalence of intracranial hemorrhage after blunt head trauma as has been reported for warfarin.

How does this improve population health?

If validated, these findings suggest that previously established guidelines regarding fall- and trauma-risk assessment and warfarin use could be applied to dabigatran.

### Outcome Measures

The primary outcome of interest was the presence of intracranial hemorrhage on initial cranial CT as interpreted by a board-certified/eligible radiologist. The presence of intracranial hemorrhage as well as the type and extent of injury were directly abstracted from the final radiology report. Specific treatments including attempted reversal (defined as either administration of prothrombin complex concentrates, plasma, recombinant factor VIIa, or dialysis for the specific purpose of reversing the effects of dabigatran), neurosurgical intervention, and hospital length of stay (in days) were abstracted. Final disposition as reported on hospital discharge summary was also recorded.

### Primary Data Analysis

We reported normally distributed continuous data as the mean with standard deviations (SD), and we described ordinal or non-normally distributed continuous data as medians with interquartile (25%–75%) ranges (IQR). Interrater reliability is reported as Cohen’s kappa. We performed all statistical analyses using STATA 14.1 (STATA Corp., College Station, TX).

## RESULTS

### Characteristics of Study Participants

During the study period there were a total of 98 ED visits by 85 patients taking dabigatran during which cranial CT was performed. Of the 98 visits, 33 met inclusion/exclusion criteria and were included in the final study population. We excluded 49 visits because there were no history or physical exam findings of trauma. Eleven patients with traumatic injuries were excluded because they did not sustain head trauma. We excluded an additional five patients were excluded because they were transferred from outside facilities (Figure).

Baseline characteristics for the study population are provided in Table 1. Mean age was 74.8 years (SD 11.2, range 55–91). The most common cause of injury was ground-level fall (n = 22, 66.7%). Initial GCS scores were all either 14 (n=4) or 15 (n=29). A total of 19 patients (57.6%, 95% CI 39.2–74.5%) were admitted to the hospital with a median ISS of 6 (IQR 3–9). Treatment and outcomes are reported in Table 2. Initial INR measurements were available in 24 patients, and activated partial thromboplastin time (aPTT) measurements were available in 23 patients. Median INR was 1.2 (IQR 1.1–1.3) and mean aPTT was 38.8 (SD 12.9, range 14.3–66.1). Thirteen (54%, 95% CI [33–74%]) patients had INR measurements greater than the upper limit of normal (1.18) for the study site, and 13 (57%, 95% CI [34–77%]) had aPTT measurements greater than the upper limit of normal (36.7) for the study site.

One patient was found to have an intracranial hemorrhage on cranial CT (3.0%, 95% CI [0.1%–15.8%]) following a motorcycle collision. This patient had an intra-ventricular hemorrhage on CT and was also noted to have pelvic, cervical spine, and rib fractures. After a hospital stay of 19 days this patient was discharged to a skilled nursing facility. There were no patients that required neurosurgical intervention (0%, 95% CI [0–8.7%]).

Patients also suffered the following major injuries: spinal fractures (n=3), long bone fractures (n=2), rib fractures (n=2), and pelvic fracture (n=1). The median hospital length of stay was two days (IQR 1–7 days) for those admitted, and one in-hospital death was identified following infectious complications after a hospital stay of 19 days.

Reversal of anticoagulation was attempted in two patients. One patient received both 4-factor activated prothrombin complex concentrate (FEIBA^©^) and dialysis following a cervical spine fracture. The second patient received a combination of fresh frozen plasma, FEIBA^©^, and dialysis for intracranial hemorrhage, pelvic fractures, rib fractures, and a cervical spine fracture.

### Interrater reliability

To measure interrater reliability of data abstraction, 20 patients had duplicate abstraction for presence of trauma, intracranial hemorrhage on cranial CT, and initial GCS score. There was perfect agreement for presence of trauma and the presence of intracranial hemorrhage on cranial CT (kappa=1.0, 95% CI [0.63–1]). There was excellent agreement for initial GCS (kappa=0.78, 95% CI [0.30–1.0]).

## DISCUSSION

In this study we found that the risk of intracranial hemorrhage after blunt head trauma in patients taking dabigatran is similar to the prevalence previously reported for warfarin.^16^ Furthermore, we have demonstrated that many patients who report taking dabigatran at the time of ED presentation have normal clotting parameters (PT and aPTT), suggesting either non-compliance or a poor correlation between dabigatran effects and currently available anticoagulant tests. There were a significant number of patients who required intensive care unit monitoring. However, a majority of patients were discharged home and the mortality rate was low.

The relatively low prevalence of intracranial hemorrhage in patients initially presenting to our institution is surprising. Given the large CI, it may be true that a larger sample size would reveal a higher “true” prevalence. It could also partially be explained by the fact that a majority of patients had ground-level falls and low ISS. Previous studies of patients with mild head injury found a prevalence of intracranial hemorrhage of 4.3% if the patient was on pre-injury warfarin.^6^

Dabigatran is the first of several DOACs to receive approval by the FDA. It exerts its action by directly binding to thrombin. When it first entered the market it was touted for its benefits of not requiring routine lab testing and very few drug-drug interactions. Initial experience with hemorrhaging patients on dabigatran raised concerns regarding the lack of ability to accurately determine the degree of anticoagulation and unclear method of reversal.^12^,^17^ Fortunately, with the recent approval of idarucizumab, a dabigatran-specific antibody fragment, the ability to treat bleeding patients on dabigatran is likely to improve.^18^ The impact of this agent on patient outcomes or patient need for dialysis, however, is still unknown.

Bleeding risk remains the main concern associated with anticoagulant use. The Randomized Evaluation of Long-Term Anticoagulant Therapy (RE-LY) study upon which initial approval of dabigatran was based demonstrated that patients taking dabigatran have a lower risk of major bleeding than patients taking warfarin.^2^ In this study of 18,113 patients, 46 cases of traumatic intracranial hemorrhage were identified, 24 in patients on warfarin and 22 in the dabigatran groups.^19^ However, this study did not report the number of patients with blunt head trauma without resultant intracranial hemorrhage. Additional case reports of intracranial hemorrhage have been reported in the literature with some raising concern that pre-injury dabigatran use could result in significant hemorrhage expansion.^17^ In an animal model, Schaeffer et al. analyzed the size of hemorrhage produced in a standardized fashion in rats given pre-injury dabigatran or warfarin. This study showed smaller hematoma size in rats given dabigatran; however, no differences in neurologic outcomes at day 21 were identified.^20^

To date, only one human study has evaluated the prevalence of intracranial hemorrhage in patients on dabigatran. This study included elderly patients (>65 years) with ground-level falls who were admitted to a trauma service. The authors compared the intracranial hemorrhage prevalence in the dabigatran group to patients on warfarin and found no difference (13.6% (warfarin) vs. 8.2%(dabigatran)).^21^ However, this study excluded patients discharged from the ED and included patients transferred to the study site. Both of these factors could result in overestimation of the risk of intracranial hemorrhage after trauma.

## LIMITATIONS

This was a retrospective analysis, which has inherent limitations. We attempted to minimize potential bias from the design by adhering to best practice guidelines for retrospective reviews.^14^ However, because all abstractors were part of the initial study design, they were not blinded to the study hypothesis. The potential bias that this lack of blinding could introduce is mitigated by the fact that the primary study outcome is an objective finding on cranial CT. We also demonstrate excellent interrater reliability enhancing the reliably of the data.

We selected only those patients undergoing cranial CT scanning in the ED, resulting in an inability to identify patients with minor trauma treated without cranial imaging. We used this criterion as it gave us the greatest chance of identifying all head trauma patients. It is normal practice in the study site’s ED to image patients with head trauma who are on anticoagulation medications.^16^ We believe that the bias this decision introduced might have increased the reported prevalence of intracranial hemorrhage due to exclusion of patients at lowest risk. However, there is the possibility that there were patients who did not receive initial imaging and subsequently presented to other centers with traumatic intracranial hemorrhage.

Due to the fact that this was a single-center study, we were unable to evaluate for later presentations for delayed bleeding. Our methods would have discovered any patients that re-presented to the ED during the study period, but could not evaluate for patients who later presented to alternate sites. The single-center nature of this study also resulted in a small sample size, which limits the generalizability of our results.

Finally, because we are a tertiary-care referral center, we excluded patients transferred from outside facilities to allow for determination of a “true” prevalence of intracranial hemorrhage in patients presenting to the ED. Two of the five excluded patients were transferred to the study site because they had intracranial hemorrhages. Neither of these patients died during hospitalization at the study site and only one required neurosurgical intervention. Including these patients would have falsely increased the reported prevalence of intracranial hemorrhage on CT.

## CONCLUSION

In our study, intracranial hemorrhage after blunt head trauma in patients on pre-injury dabigatran was rare. The incidence in our study is similar to previous reports for patients on warfarin, although the wide confidence interval and different methodology make direct comparison difficult. Further studies are needed to determine if the prevalence of intracranial hemorrhage seen in our patient population is true for a larger patient cohort in more diverse clinical settings.

## Figures and Tables

**Figure:**
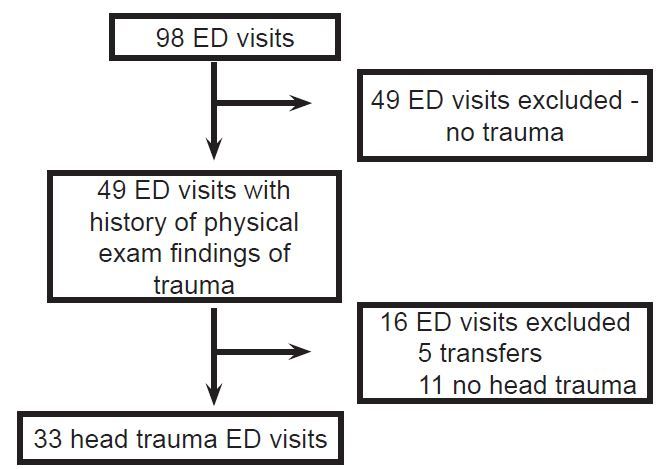
Flow of patients in a study of the prevalence of intracranial hemorrhage after blunt head trauma for patients on dabigatran. *ED*, emergency department.

**Table 1:** Demographics and injury characteristics.

	No.	%
Sex		
Female	18	54.5
Male	15	45.5
Indication for dabigatran		
Atrial fibrillation	27	81.8
Deep venous thrombosis	2	6.1
Pulmonary embolism	1	3.0
Unknown	1	3.0
Other	2	6.1
Mechanism of injury		
Assault	1	3.0
Auto vs pedestrian	1	3.0
Ground-level fall	22	66.7
Motorcycle collision	2	6.1
Motor vehicle collision	3	9.1
Other	4	12.1
ICH	1	3.0

*ICH*, intracranial hemorrhage.

**Table 2:** Treatment and outcomes of patients with blunt head trauma who were taking dabigatran at time of arrival to Level I trauma center.

	No.	%
Reversal Agent		
None	30	93.9
aPCC	2	6.1
Transfused with Plasma	1	3.0
Dialysis	2	6.1
PRBC Transfusion	1	3.0
ED disposition		
Discharged from the ED	14	42.4
Ward	5	15.2
Telemetry	6	18.2
ICU	8	24.2
Final Hospital/ED disposition		
Died	1	3.0
Skilled Nursing facility	5	15.2
Home	24	72.7
Other	3	9.1

*aPCC*, activated 4-factor prothrombin complex concentrate; *PRBC*, packed red blood cells; *ED*, emergency department
